# A Rare Presentation of a Duodenal Neuroendocrine Tumor

**DOI:** 10.7759/cureus.33747

**Published:** 2023-01-13

**Authors:** Aboud Kaliounji, Sami Alkoutami, Michael Farraj, Haya Kaliounji, Kristen L Farraj

**Affiliations:** 1 Internal Medicine, State University of New York (SUNY) Downstate Medical Center, Brooklyn, USA; 2 Internal Medicine, St. George's University School of Medicine, St. George's, GRD; 3 Internal Medicine, Nassau University Medical Center, East Meadow, USA; 4 Internal Medicine, University of Colorado Anschutz Medical Campus, Aurora, USA

**Keywords:** small bowel, gastroenterology tumor, neuroendocrine tumor, gist, duodenum

## Abstract

Neuroendocrine tumors (NETs), which are a rare type of tumor, are defined as epithelial cells with predominantly neuroendocrine differentiation and consist of a spectrum of tumors emerging from stem cells throughout the body and can occur anywhere in the body. While they are rare, the incidence over the past few decades has increased. Here we present a case of a 64-year-old female who was incidentally found to have a duodenal neuroendocrine tumor. The patient initially presented to the emergency department secondary to syncope and collapse. During her trauma evaluation, an incidental lobulated soft tissue mass inferior to the distal stomach was seen on complete computed tomography (CT) scans. The surgery team was consulted for resection of the mass and an octreotide scan was performed prior to resection to further evaluate the mass and to check for any signs of metastatic disease. The octreotide scan demonstrated intense radiotracer accumulation within the duodenal mass consistent with a neuroendocrine tumor and no areas of abnormal radiotracer accumulation suspicious for metastatic disease. Pathology of the resected mass was positive for a well-differentiated neuroendocrine tumor with an organoid pattern and homogenous oval-round neoplastic cells with a salt-pepper nuclear and pseudo glandular arrangement that was well-circumscribed and partially encapsulated with negative margins. Immunohistochemistry was positive for AE ⅓, CD56, Synaptophysin, and chromogranin and negative for CD117, DOG-1, CD34, and CD45. The prevalence of NETs has increased over the years due to the improvement in diagnostic tools, such as upper gastrointestinal endoscopy. In addition to the fact that the duodenum is a rare location for such tumors, neuroendocrine tumors are also typically found in those under 50 years old. However, our patient was found to have both a duodenal mass and was over the age of 50 at the time of presentation and diagnosis. To date, a consensus on a conclusive treatment of duodenal NETs (D-NETs) has not been reached. This case brings to light the importance of further research in diagnosing and treating neuroendocrine tumors and also raises awareness for clinicians to have this in their differential.

## Introduction

Neuroendocrine tumors (NETs) are rare types of tumors, only contributing to 0.5% of all malignancies, and are found predominantly in females under the age of 50 [1]. NETs are defined as epithelial cells with predominantly neuroendocrine differentiation. Neuroendocrine differentiation refers to cells that develop from both the nervous system and endocrine, forming heterogeneous tumors [2]. This cellular differentiation allows NETs to share similarities while still holding some unique functions based on the tumor location. The primary locations for NETs are the gastrointestinal tract (62-67%) and pulmonary tract (22-27%) [1]. Further analysis shows duodenal NETs (D-NETs) only contribute to 2-3% of all gastrointestinal tract NETs [3]. There are five unique subtypes of duodenal NETs, which include: duodenal gastrinoma, duodenal somatostatinoma, nonfunctioning duodenal NET, duodenal gangliocytic paraganglioma, and poorly differentiated neuroendocrine carcinomas [4]. The prevalence of NETs has increased over the years due to the improvement in diagnostic tools, such as upper gastrointestinal endoscopy [4]. 

While most NETs are asymptomatic and are incidental findings on imaging, treatment plans are decided based on tumor size, location, grade, stage, and tumor type [5]. In 2005, Hoffmann et al. found that NETs, specifically duodenal NETs, could be resected endoscopically if under 1 cm, while those larger are typically resolved through surgical resection [5]. Regardless of this assessment, there is no consensus regarding the ideal treatment strategy for duodenal NETs [5]. ​​In this clinical case that was previously presented as a poster abstract at the 2022 American College of Gastroenterology (ACG) Annual Conference on October 25th, 2022, we describe a case of an elderly female who was found to have a duodenal neuroendocrine tumor.

## Case presentation

**Figure 1:**
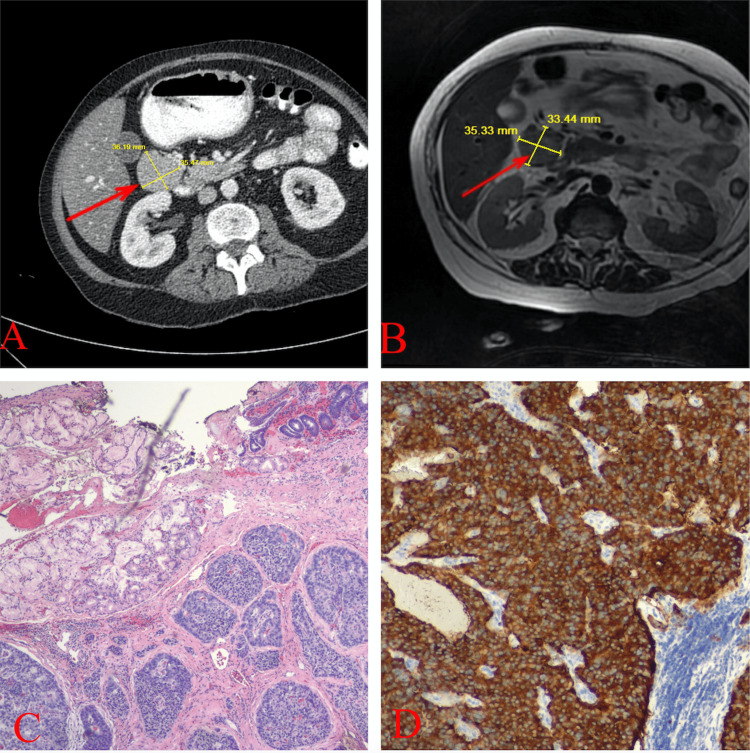
A) CT abdomen and pelvis with contrast displaying a lobulated soft tissue mass inferior to the pylorus measuring approximately 3.0 x 3.3 x 3.5 cm with possible connection with the adjacent bowel (red arrow). B) MRI of the abdomen showing a paraduodenal mass (red arrow). C) Histological slide demonstrating the surface duodenal mucosa and underlying organoid pattern of neuroendocrine tumor. D) Immunohistochemistry slide demonstrating the positivity of synaptophysin of the neoplastic cells.

A 64-year-old female with a past medical history of hypertension, glaucoma, and alcohol abuse was initially admitted to the cardiac service for evaluation of syncope and collapse with head trauma and loss of consciousness. Admission labs are shown in Table 1. During the trauma team evaluation, secondary to the fall, an incidental lobulated soft tissue mass inferior to the distal stomach was seen on complete Computed Tomography (CT) scans. The CT abdomen with contrast showed no evidence of acute traumatic injury to the chest, abdomen, or pelvis, an enlarged liver measuring 18 cm in craniocaudal dimension with diffuse fatty infiltration, no focal hepatic lesion, no biliary ductal dilatation, and a lobulated soft tissue mass inferior to the pylorus measuring approximately 3.0 x 3.3 x 3.5 cm with possible connection with the adjacent bowel but no evidence of bowel obstruction, bowel wall thickening or intraperitoneal free air (Figure 1A). The gastroenterology team was consulted for further evaluation of the mass and an esophagoduodenoscopy (EGD) was performed which was positive for Los Angeles (LA) Classification Grade B esophagitis, external compression was noted in the duodenal bulb, gastric and duodenal erosions, erythematous mucosa suggesting gastritis, and portal hypertensive gastropathy. Gastric biopsies performed during the EGD were positive for mild chronic gastritis but no helicobacter pylori-like organisms in both the antrum or the body. Tumor markers, which included alpha-fetoprotein (AFP), carcinoembryonic antigen (CEA), and cancer antigen 19-9 (CA 19-9), were obtained and were all negative. A right upper quadrant abdominal ultrasound (US) was then performed which again demonstrated a lobulated soft tissue mass inferior to the medial right hepatic lobe adjacent to the distal stomach/proximal duodenum, hepatic steatosis, no cholelithiasis, and/or biliary ductal dilatation but a magnetic resonance imaging (MRI) was recommended for further evaluation. The next day the MRI of the abdomen with and without contrast was performed which was positive for a paraduodenal mass of unknown etiology and hepatic steatosis (Figure 1B). 

**Table 1:** Admission laboratory results ALT: alanine aminotransferase, AST: aspartate aminotransferase

	Lab Results	Reference
Hemoglobin	11.6 g/dL	12.1-15.1 g/dL
Hematocrit	31.9%	36-48%
Mean Corpuscular Volume	99.4 fL	80-100 fL
White blood count	7.02 x 10^9/L	4.5-11 x 10^9/L
Potassium	3.1 mmol/L	3.6-5.2 mmol/L
Lipase	309 U/L	0-160 U/L
Amylase	228 U/L	40-140 U/L
ALT	113 U/L	4-36 U/L
AST	75 U/L	8-33 U/L
Ethanol level	262 mg/dL	< 50mg/dL
Vitamin D 25-hydroxy	25ng/mL	30-50ng/mL

The surgery team was consulted for resection of the mass. Prior to the resection of the mass, an octreotide scan was performed to further evaluate the mass and to check for any signs of metastatic disease. The octreotide scan demonstrated intense radiotracer accumulation within the duodenal mass consistent with a neuroendocrine tumor and no areas of abnormal radiotracer accumulation suspicious for metastatic disease. She was subsequently scheduled for elective resection of the tumor. Intraoperatively, a horizontal incision was made about 3 cm superior to the umbilicus with a skin knife, which was carried through subcutaneous tissue with electrocautery and the peritoneal cavity was entered. Some omental adhesions to the anterior abdominal wall were encountered and divided. The mass was immediately palpable in the duodenum. The duodenum was separated from its lateral attachments and the tumor was found to be attached to a segment of D2 on the anterior wall. It appeared adherent through the wall with a fairly narrow stalk. The right gastroepiploic vessels were divided and the mass and segment of the anterior wall of D2 was excised. This segment came back negative for gastrointestinal stromal tumor (GIST) tumor. The duodenal defect was closed, the abdomen was then irrigated, Jackson-Pratt (JP) drains were placed, and the skin was closed. Occlusive dressings were placed, the patient was extubated in the operating room, and she was taken to the post anesthesia care unit (PACU) in stable condition. The estimated blood loss was 200 mL. Pathology was positive for a well-differentiated neuroendocrine tumor with an organoid pattern and homogenous oval-round neoplastic cells with salt-pepper nuclear and pseudoglandular arrangement that was well-circumscribed and partially encapsulated with negative margins. Immunohistochemistry was positive for AE ⅓, CD56, Synaptophysin, and chromogranin and negative for CD117, DOG-1, CD34 and CD45 (Figure 1C, 1D). Upon postoperative follow up the patient stated she was doing well, that the pain had been well controlled, and that she had been able to ambulate without difficulty. 

## Discussion

Neuroendocrine neoplasms consist of a spectrum of tumors emerging from stem cells throughout the body and can occur anywhere in the body. While they are rare, the incidence over the past few decades as well as the survival rate has increased, indicating that they are more prevalent than previously thought and better diagnostic tools are being implemented to detect them [6]. The clinical presentation of those neoplasms is very similar to other gastrointestinal (GI) tract tumors, making it challenging to specifically detect those types of tumors clinically. These manifestations include abdominal pain, diarrhea, lower GI bleeding, weight loss, etc [3]. They are most commonly found in the rectum, lungs, small intestines, and appendix [6]. In fact, the GI tract accounts for almost 50% of all neuroendocrine tumors [3]. The duodenum is a less common location for such tumors, accounting only for 4% of GI tumors, which is the case of our patient [7]. 

In 2015, Fitzgerald et al. stated that there has been an increase in the incidence of D-NETs as well as gastroenteropancreatic neuroendocrine tumors (GEP-NETs) [8]. In their study, Fitzgerald et al. identified 1,258 patients diagnosed with carcinoid tumors from 1983 to 2010. They were divided into two groups; the first group included those diagnosed between 1983 and 2005, and the second, those diagnosed between 2005 and 2010. There was a marked increase in D-NET incidence from 0.27 per 100,000 in 1983 to 1.1 per 100,000 in 2010 (p-value < 0.1) [8]. In fact, the incidence rate in the United States is 0.19 per 100,000 [6]. On the other hand, England and Japan have seen a lower prevalence with only 0.04 per 100,000 and 0.17 per 100,000, respectively [9,10].

Because almost 90% of the D-NETs are non-functional, the majority of them are incidentally detected on imaging or endoscopy [5]. Tissue biopsy and EGD are in fact the most common method to reach a diagnosis. While imaging is useful to detect the tumor, histopathology is commonly used to identify the type of tumor. They are predominantly found in the first and second portions of the duodenum, with the periampullary region accounting for 20% of the cases [11]. They are usually small and solitary, with around 75% of them smaller than 20mm [11]. Metastasis to the lymph nodes and liver occurs in 40%-60% and 10%, respectively, even though the majority of D-NETs are limited to the mucosa and submucosa [5,12]. 

D-NETs are classified into three grades: G1, G2, and G3, depending on Ki-67 index and the number of mitotic figures. G1 corresponds to a Ki-67 index of less than 2% and/or a number of mitotic figures less than two. G2 corresponds to a Ki-67 index between 3 and 20% and/or a number of mitotic figures between two and 20. G3 corresponds to a Ki-67 index above 20% and/or a number of mitotic figures above 20 [13]. The overall prognosis of this disease depends on the classification, tumor type, location, size, staging, and grading. For instance, G1-NET has a very good prognosis. The five-year survival rate is 80%-85% in people with a well-differentiated D-NET [14]. 

To date, a consensus on a conclusive treatment of D-NETs has not been reached. However, the management is based on tumor type, stage, location, size, and histological grade. Today, resection of the tumor appears to be the most radical treatment. However, whether it is surgical or endoscopic removal depends on its size. Because the pathogenesis of the disease is yet to be understood, this case brings to light the importance of further research in diagnosing and treating neuroendocrine tumors and also raises awareness for clinicians to have this in their differential.

## Conclusions

NETs are rare types of tumors that arise from stem cells and can occur anywhere in the body. While the majority of neuroendocrine tumors are asymptomatic, their clinical presentation is very similar to other GI tumors. Thus, it is imperative for clinicians to include this rare malignancy in their differential in patients presenting with broad symptoms. With its increased incidence over the past few decades due to improved diagnostic studies, more research is needed to further investigate the risk factors associated with it, the prognosis, as well as treatment options other than surgical removal. 
